# Cytomegalovirus Infection Causes an Increase of Arterial Blood Pressure

**DOI:** 10.1371/journal.ppat.1000427

**Published:** 2009-05-15

**Authors:** Jilin Cheng, Qingen Ke, Zhuang Jin, Haibin Wang, Olivier Kocher, James P. Morgan, Jielin Zhang, Clyde S. Crumpacker

**Affiliations:** 1 Division of Infectious Diseases, Beth Israel Deaconess Medical Center, Harvard Medical School, Boston, Massachusetts, United States of America; 2 Division of Cardiology, Beth Israel Deaconess Medical Center, Harvard Medical School, Boston, Massachusetts, United States of America; 3 Division of Allergy, Beth Israel Deaconess Medical Center, Harvard Medical School, Boston, Massachusetts, United States of America; 4 Department of Pathology, Beth Israel Deaconess Medical Center, Harvard Medical School, Boston, Massachusetts, United States of America; 5 Cardiovascular Center, Caritas St. Elizabeth's Medical Center, Tufts University School of Medicine, Boston, Massachusetts, United States of America; Oregon Health & Science University, United States of America

## Abstract

Cytomegalovirus (CMV) infection is a common infection in adults (seropositive 60–99% globally), and is associated with cardiovascular diseases, in line with risk factors such as hypertension and atherosclerosis. Several viral infections are linked to hypertension, including human herpes virus 8 (HHV-8) and HIV-1. The mechanisms of how viral infection contributes to hypertension or increased blood pressure are not defined. In this report, the role of CMV infection as a cause of increased blood pressure and in forming aortic atherosclerotic plaques is examined. Using *in vivo* mouse model and *in vitro* molecular biology analyses, we find that CMV infection alone caused a significant increase in arterial blood pressure (ABp) (*p*<0.01∼0.05), measured by microtip catheter technique. This increase in blood pressure by mouse CMV (MCMV) was independent of atherosclerotic plaque formation in the aorta, defined by histological analyses. MCMV DNA was detected in blood vessel samples of viral infected mice but not in the control mice by nested PCR assay. MCMV significantly increased expression of pro-inflammatory cytokines IL-6, TNF-α, and MCP-1 in mouse serum by enzyme-linked immunosorbent assay (ELISA). Using quantitative real time reverse transcriptase PCR (Q-RT-PCR) and Western blot, we find that CMV stimulated expression of renin in mouse and human cells in an infectious dose-dependent manner. Co-staining and immunofluorescent microscopy analyses showed that MCMV infection stimulated renin expression at a single cell level. Further examination of angiotensin-II (Ang II) in mouse serum and arterial tissues with ELISA showed an increased expression of Ang II by MCMV infection. Consistent with the findings of the mouse trial, human CMV (HCMV) infection of blood vessel endothelial cells (EC) induced renin expression in a non-lytic infection manner. Viral replication kinetics and plaque formation assay showed that an active, CMV persistent infection in EC and expression of viral genes might underpin the molecular mechanism. These results show that CMV infection is a risk factor for increased arterial blood pressure, and is a co-factor in aortic atherosclerosis. Viral persistent infection of EC may underlie the mechanism. Control of CMV infection can be developed to restrict hypertension and atherosclerosis in the cardiovascular system.

## Author Summary

Cytomegalovirus (CMV) infection is associated with cardiovascular diseases. The exact mechanisms, however, remain to be defined. Using both mouse model and cell culture analyses, we find that CMV infection alone causes an increase in blood pressure. Additionally, CMV infection augments the increased blood pressure induced by a high cholesterol diet. CMV infection alone, however, does not cause atherosclerosis in aortas. CMV infection along with a high cholesterol diet, however, causes the classic atherosclerotic plaque formation in the main artery connected to the heart. Further studies show that CMV infection induces renin and angiotensin II (Ang II) expression in blood and in vessel cells, in a persistent infection manner. An increased expression of renin and Ang II has been known to cause an increase in blood pressure or hypertension in humans. Expression of viral genes and viral persistent infection of blood vessel endothelial cells resulting in an increased expression of inflammatory cytokines, including renin and Ang II, may underpin the molecular mechanism by which CMV infection induces an increase in blood pressure.

## Introduction

Human cytomegalovirus (HCMV) is a member of the herpes virus family, and HCMV infection is ranked as one of the most common infections in adults, with the seropositive rates ranging from 60–99% globally. Once acquired, the infection persists lifelong and may undergo periodic reactivation. The infection with HCMV is associated with cardiovascular diseases, and some countries have reported low rates of HCMV seropositivity and a high incidence of atherosclerosis [1]–[4]. Additionally, several virus infections are associated with hypertension or an increase of blood pressure, including human herpesvirus 8 (HHV-8) and HIV-1 in primary pulmonary hypertension [5]–[7]. The mechanisms underlying how viruses contribute to hypertension have not been identified. In a mouse model of pulmonary hypertension, a recent paper has explored the mechanism of pulmonary artery muscularization and arterial remodeling by inflammation and the Th2 immune response [8]. Clinical isolates of HCMV have been shown to infect endothelial cells, and the presence of HCMV antigens in endothelial cells triggers inflammation and immune response via secretion of CXC chemokines and recruiting neutrophils, which are infected by HCMV in the process of neutrophil transendothelial migration [9].

HCMV infection is also implicated in atherosclerosis, as in the report that HCMV-seropositive individuals have endothelial dysfunction and an increased atherosclerosis burden [10]. The use of the anti-CMV drug, ganciclovir, appears to lower heart transplant related atherosclerosis [11]. Studies in apolipoprotein E-knockout mice also show that MCMV infection increases atherosclerosis [12]. Other clinical studies, however, have not found an association between HCMV infection and vascular atherosclerosis [13]–[17]. It remains an important investigational subject, therefore, to define the role of CMV in vascular injury and atherosclerosis. This could also be mediated by CMV inducing vascular injury and causing hypertension, which serves as a co-factor to interact with other factors to induce atherosclerosis. As atherosclerosis is a complicated event with lipid metabolism, genetic factors and inflammatory pathways obviously playing crucial roles, it is important to define that a common widespread virus, such as CMV, might initiate atherosclerosis or inflammatory response resulting in vascular injury. This has the potential to lead to new treatments for vascular disease directed at the antiviral therapy of CMV or prevention by a vaccine against CMV. Furthermore, distinguishing the role of CMV infection in vascular cells and atherosclerosis adds to elucidating the mechanism of CMV associated cardiovascular diseases (CV), since CMV infection is reported as a primary factor and directly linked to CV as in myocardial infarction, stroke, coronary restenosis, or CV death [18]–[20].

In this study, we have employed *in vivo* and *in vitro* experimental systems and defined that MCMV infection alone results in a significant increase of blood pressure. Molecular biology analyses show that MCMV infection stimulated pro-inflammatory cytokine expression, which have previously been shown to play a role in an increase of blood pressure [21]–[23]. Specifically, we have identified that CMV infection induced expression of renin in an infection dose responsive manner in mouse renal cells and in human vascular endothelial cells. Additionally, an increased angiotensin II (Ang II) level was detected in mouse serum and in arterial blood vascular tissues after MCMV infection. This is of great interest, since renin is known as a rate limiting protein of Renin-Ang-II system (RAS) and Ang II is the effector peptide that directly binds to blood vessels, causes vasoconstriction and leads to systemic hypertension in humans [24]–[27]. Our studies have defined that CMV infection alone leads to an increase in blood pressure, whereas CMV acts as a co-factor, along with high cholesterol diet to induce atherosclerosis in the mouse aorta. A persistent CMV infection of EC and an increased pro-inflammatory cytokine expression, including renin and AngII, may underlie the molecular mechanism by which CMV infection induced an increase of blood pressure.

## Results

### MCMV infection significantly increases blood pressure *in vivo*

To identify the relationship between CMV infection and an increase of blood pressure, we performed a trial study using 48 mice in 4 groups, to compare CMV infection with two other known risk factors in hypertension: high cholesterol diet and atherosclerosis. Mice in group 1 were infected by MCMV and fed a regular diet. Mice in group 2 were mock-infected and fed a regular diet (control group). Mice in group 3 were infected by MCMV and fed a high cholesterol diet. Mice in group 4 were mock-infected and fed a high cholesterol diet (control group).

**Figure 1:**
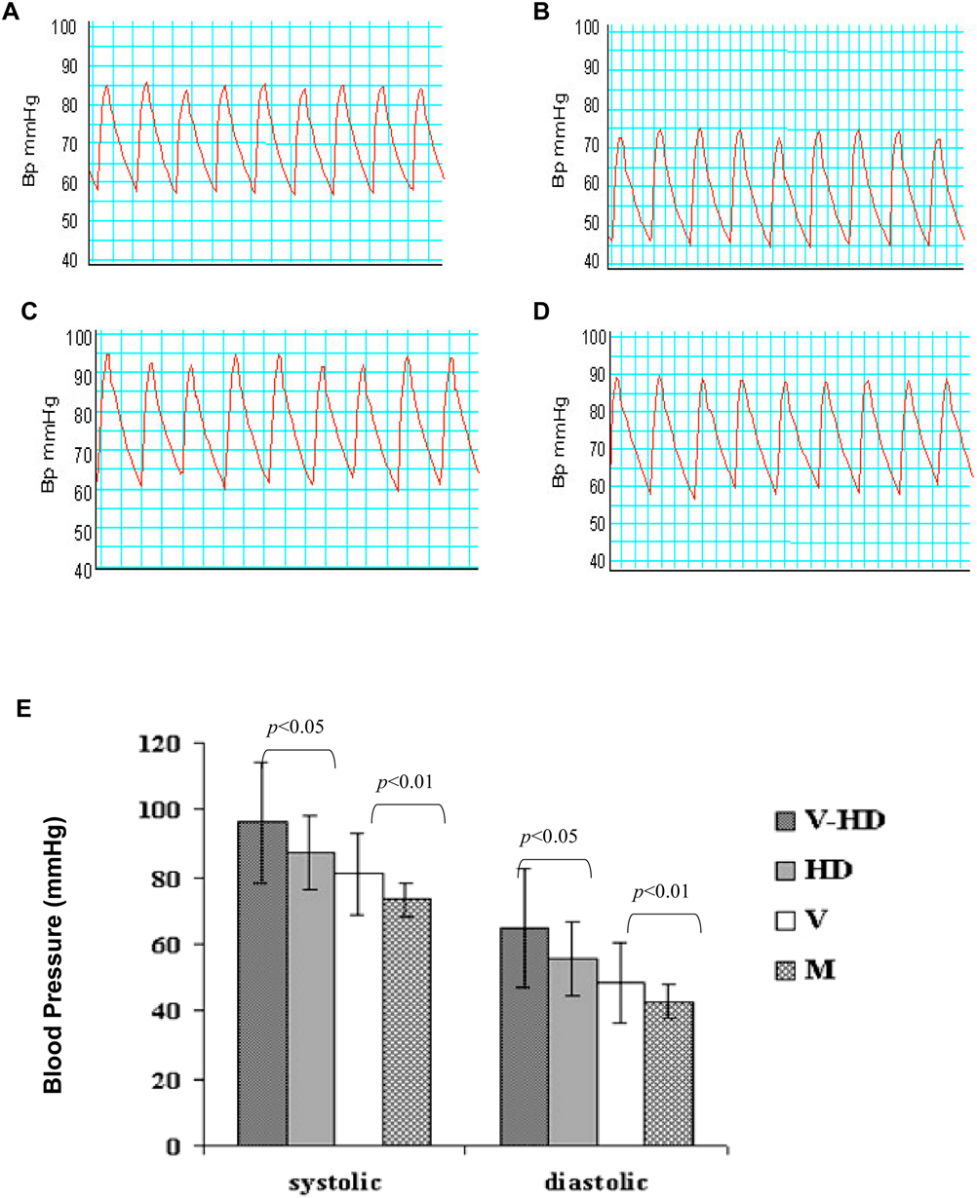
Tracing blood pressures by carotid artery catheter. A representative blood pressure tracing in a mouse from each assay group is shown (12 mice were in each group). (A) Mouse infected by MCMV. (B) Mock-infected. (C) Mouse infected by MCMV and fed a high cholesterol diet. (D) Mouse mock infected and fed a high cholesterol diet. (E) The mean value of ABp from each assay group (mmHg). V-HD: mice infected by MCMV and fed with a high cholesterol diet. HD: mice mock-infected and fed with a high cholesterol diet. V: mice infected by MCMV and fed with a regular diet. M: mice mock-infected and fed with a regular diet. Blood pressures in each group were measured at week 10 of experiment. The mean values of systolic and diastolic pressure were determined and calculated with Chart v4.1.2 software, respectively. Statistical significance between assay groups was determined with Student's *t* test. The base line blood pressure of a mouse from each assay group before MCMV infection is shown in Table S1.

The carotid blood pressure (ABp) of each mouse was measured after 6 weeks of infection by inserting a microtip catheter into the right carotid artery under anesthesia. Both systolic (each peak) and diastolic (each nadir) ABp were measured and recorded as the real time tracing [28],[29]. The experimental results show that MCMV infection alone significantly increased arterial blood pressure, compared to their counterparts in each control group (Fig. 1A–E, V-HD vs. HD; V vs. M).

### An increase in blood pressure by MCMV infection is independent of high cholesterol diet and atherosclerosis

As previously reported, high cholesterol diet increases blood pressure, and an increased blood pressure promotes atherosclerosis that further exacerbates the hypertension [30]–[33]. The effects of CMV infection, however, in relation with these two factors on an increase of blood pressure remain to be identified. In this study, we analyzed the role of MCMV infection in an increase of blood pressure, and the relationship with the two known risk factors that lead to the hypertension.

By studying the four groups of mice, we found that independent of the high cholesterol diet, MCMV infection caused a significant increase of blood pressure, since mice infected with MCMV and fed a regular diet had a significant increase in arterial blood pressure in contrast to their control counterparts (Fig. 1E, V vs. M, p<0.01). Furthermore, MCMV infection exacerbated the increase of blood pressure caused by the high cholesterol diet, as mice infected with MCMV and fed with high cholesterol diet had a significantly higher blood pressure than the mock infected control mice fed with the same diet (Fig. 1E, V-HD vs. HD, p<0.05).

**Figure 2:**
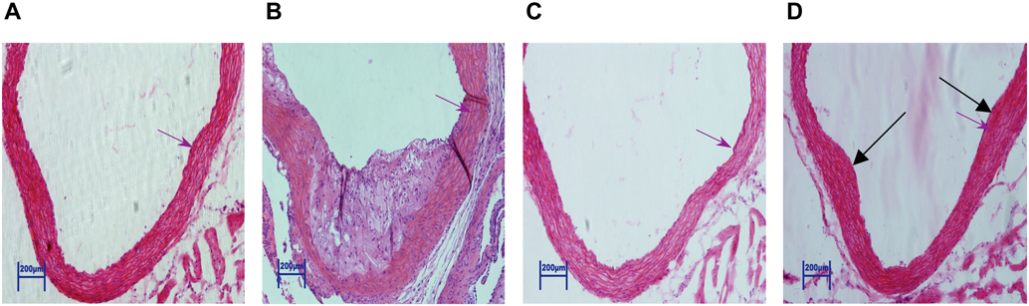
An increase in blood pressure by MCMV infection is independent of atherosclerosis. (A) Aortic root section from a mouse mock-infected and fed a high cholesterol diet showing no visible atherosclerosis. (B) Aortic root section from a mouse infected by MCMV plus fed a high cholesterol diet showing a typical atherosclerotic plaque with numerous foam cells and occasional cholesterol clefts. (C) Aortic root section from a mouse mock-infected and fed a regular diet showing no visible atherosclerosis. (D) Aortic root section from a mouse infected by MCMV and fed a regular diet showing intimal thickenings (arrow pointed) and no visible atherosclerosis. Each group contained 12 mice. All pictures were taken at 20× magnification, and scales are as shown in bar. The vessel wall was measured in the same position (red arrow pointed) of the aortic root of animals fed a high cholesterol diet alone (4 mm), MCMV infected plus fed on a high cholesterol diet (7 mm), fed a regular diet alone (3 mm), and MCMV infected plus fed on a regular diet (6 mm).

In determining the role of MCMV infection in atherosclerosis and an increase in blood pressure, our data show that MCMV infection and high cholesterol diet together induced atherosclerotic plaque formation in mouse aortas (Fig. 2A–D). Neither MCMV infection nor high cholesterol diet alone, however, caused atherosclerosis during the assay period. Although atherosclerotic plaque formation required the effects of both MCMV infection and high cholesterol diet, atherosclerosis was not a major factor that increased blood pressure, since MCMV infection alone significantly increased the blood pressure without the effect on atherosclerosis (Fig. 1E, V vs. M, p<0.01 and Table 1). Our results suggest that MCMV infection alone plays an important role in the increase of blood pressure *in vivo*. Furthermore, an increase in weight was not a factor in inducing high blood pressure in the context of MCMV infection, as mice fed a regular diet weighed the same, whether MCMV infected or mock-infected. Mice fed a high cholesterol diet weighed the same, regardless of MCMV infected or not (Fig. S1).

**Table 1:** Atherosclerotic plaque formation in mouse aorta.

Experimental group	Mice	Mouse with atherosclerotic plaques	*P*
MCMV (V)	12	0	
Mock-infected (M)	12	0	
MCMV + High cholesterol diet (V+HD)	12	3	
**vs**			**<0.01**
High cholesterol diet (HD)	12	0	

### MCMV infection increased pro-inflammatory cytokine levels in mouse blood

Others have previously reported that stimulation of pro-inflammatory cytokine expression increases blood pressure [21]–[23]. To identify whether MCMV infection had triggered expression of pro-inflammatory cytokines and contributed to the increase of blood pressure, we studied the levels of three cytokines in mouse blood by cytokine specific ELISA.

**Figure 3:**
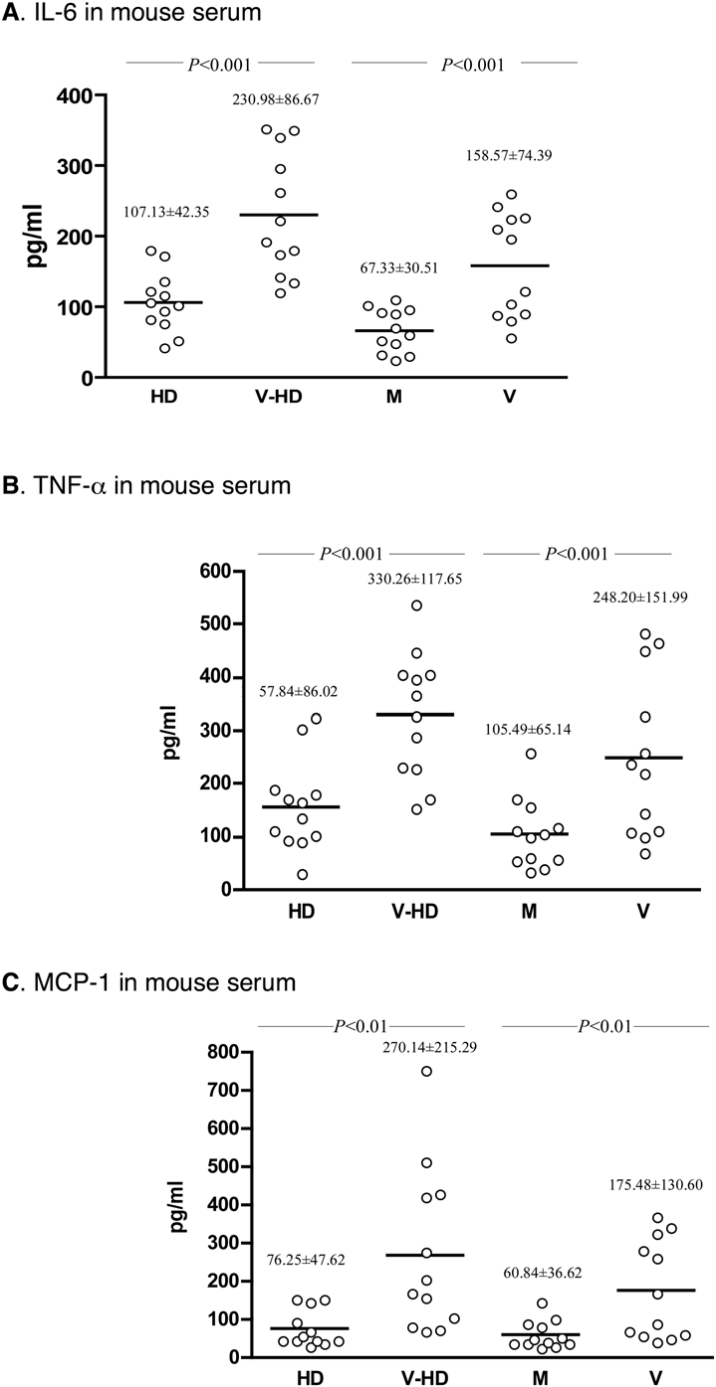
MCMV infection stimulated expression of pro-inflammatory cytokines in mouse serum. The systemic changes on IL-6, TNF-α and MCP-1 levels in 4 experimental groups were determined by ELISA (each open circle represents a mouse). (A) IL-6 in mice infected with MCMV was significantly higher than in the mock-infected groups fed with either diet (*P*<0.001). (B) TNF-α level in mice infected with MCMV was significantly higher than in the mock-infected groups fed with either diet (*P*<0.001). (C) MCP-1 level in mice infected with MCMV was significantly higher than in the mock-infected groups fed with either diet (*P*<0.01). Statistical significance between assay groups was determined with Student's *t* test, and each group contained 12 mice.

The serum levels of IL-6, TNF-α and MCP-1 were significantly increased in mice infected with MCMV than in mice mock-infected (Fig. 3A–C; *P*<0.001, <0.001 and <0.01, respectively). Specifically, diet had no impact on expression of these cytokines in mice infected by MCMV, in comparison to the control mice (*P*>0.05). These results indicate that MCMV infection triggered a significant increase of pro-inflammatory cytokine expression in mouse blood, which might participate in the blood pressure increase induced by CMV infection *in vivo*.

### MCMV infection stimulates expression of renin

Besides determining the increased expression of inflammatory cytokines, we examined whether CMV infection triggered factors that are known to regulate blood pressure. Renin is the first and a step-limiting protein of RAS. RAS plays a central role in regulating blood pressure. An increased RAS activity leads to an increase of blood pressure or systemic hypertension [24]–[27].

**Figure 4:**
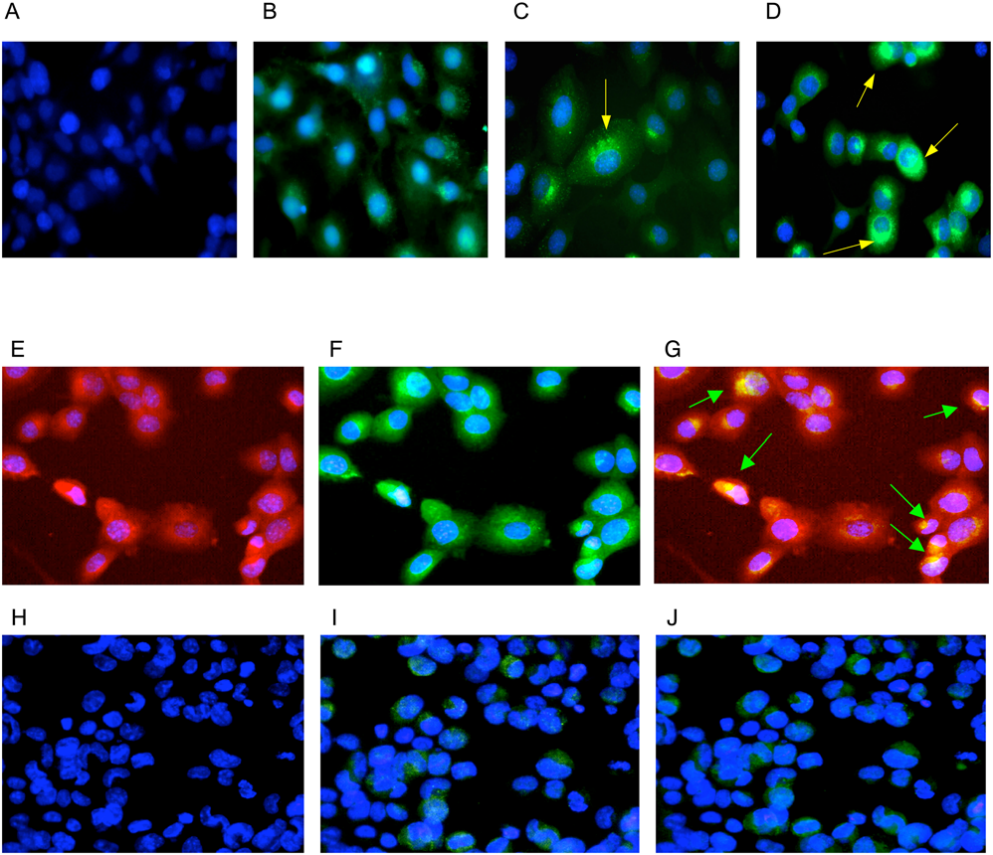
MCMV infection induced expression of renin in a dose dependent manner. (A) Antibody control. Cells were stained at day 6 post CMV infection at multiplicity of infection (MOI) = 10, with a non-specific IgG as the first antibody. (B) Mock infection control (MOI = 0). Cells were stained at day 6 post mock infection by renin specific IgG as the first antibody. Renin was detected at a low level, with positive fine granules suffused in the cytoplasm, as As4.1 cells have a basal level expression of renin from Ren-1c locus. (C) MCMV infection in a low dose (MOI = 1). Cells were stained at day 6 post infection. Renin positive granules were big, around the nucleus. (D) MCMV infection in a high dose (MOI = 10). Cells were stained at day 6 post infection, and renin positive granules were bigger and denser, surrounding the nucleus. (E–G) Co-staining of MCMV and renin. (E) MCMV antigens were stained in red with TRITC by anti-MCMV antibodies. (F) Renin was stained in green with FTIC by anti-renin antibodies. (G) Overlay the staining of TRITC and FTIC to show MCMV and renin co-localization in cells. The yellow spots, representing the co-stain of MCMV antigen and renin, surrounded the nucleus (arrow pointed). (H–J) Controls of immunofluorescent staining. (H) Mock-infected cells stained by anti-MCMV antibodies with TRITC, no MCMV antigen was detected. (I) Mock-infected cells stained by anti-renin antibody with FTIC, only basal level expression of renin was detected. (J) Overlay of TRITC and FTIC in mock-infected cells, and no MCMV antigen signal was detected.

To define whether MCMV infection increased expression of renin, we conducted an *in vitro* study by using As4.1 cells. These cells have a basal level expression of renin from the endogenous Ren-1c locus, and As4.1 cells are kidney cells. We infected As4.1 cells by MCMV in differing multiplicities of infection (MOI), and detected renin expression by immunofluorescent microscopy. MCMV infection stimulated renin expression in cells in a MCMV infection dose dependent manner (Fig. 4A–D). Cells with mock infection only had a basal level expression of renin. Since renin generated in kidney cells is the key component of circulatory RAS, our data suggest that CMV infection stimulated RAS activity and contributed to an increase of blood pressure *in vivo*.

To further define the relationship between MCMV infection and expression of renin, we performed a co-staining assay to measure MCMV infection inducing expression of renin at a single cell level. We find that the induced renin expression occurred in MCMV infected cells only, and the MCMV antigens were co-localized with renin in the infected cells that were detected by antibodies specific to MCMV and renin, respectively (Fig. 4E–G). Mock-infected cells only had a basal level expression of renin, and no MCMV antigens were detected (Fig. 4H–J). When UV-inactivated MCMV was employed as a control in the infection, no increase in renin expression was detected (data not shown).

### MCMV infection induced expression of Ang II in mouse blood and artery tissues

**Figure 5:**
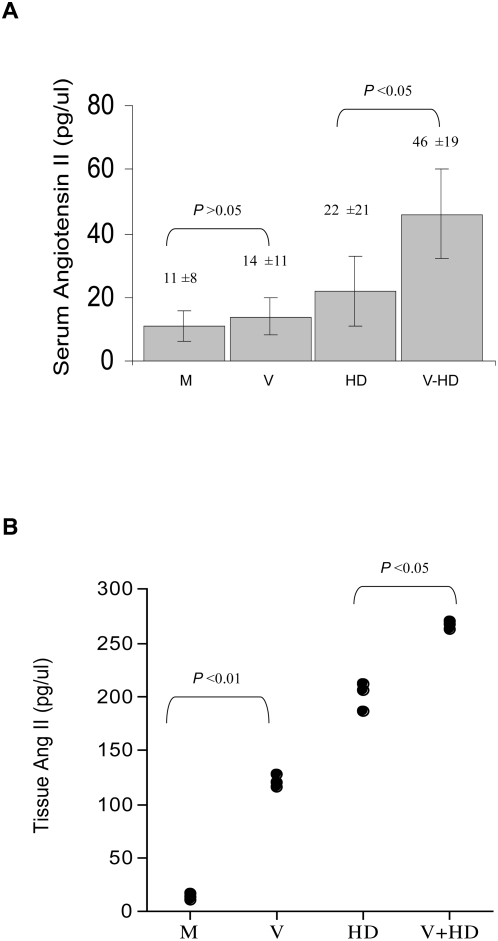
MCMV infection increased Ang II level in mouse serum and artery tissues. (A) MCMV infection increased angiotensin II (Ang II) level in mouse serum, and exacerbated the Ang II level induced by a high cholesterol diet. Each group contained 12 mice. (B) MCMV infection increased Ang II levels in aorta tissues, and exacerbated the Ang II level induced by a high cholesterol diet. Each dot represents a mouse from each assay group.

Ang II is the main effector peptide of RAS. Ang II directly binds to receptors on blood vessels and causes vasoconstriction and an increase of blood pressure [24]–[27]. To determine whether MCMV infection also changed expression of Ang II, we examined Ang II levels in mouse serum and in artery tissues. MCMV infection alone increased angiotensin II (Ang II) level in mouse serum. Additionally, MCMV infection exacerbated the increased Ang II level induced by high cholesterol diet (Fig. 5A). A similar pattern of Ang II level was detected in arterial tissue specimens. Despite a high basal level of Ang II expression in artery tissues, MCMV infection alone increased expression of Ang II in aorta samples and also exaggerated an increased expression of Ang II induced by high cholesterol diet (Fig. 5B). These results suggest that CMV infection induced renin and Ang II expression, and this underlies a molecular mechanism by which CMV infection causes an increase of blood pressure.

### Detection of MCMV RNA and DNA in blood vessels post infection

During the experimental period, all mice exhibited normal behavior, eating and drinking activities, without symptoms of acute MCMV infection such as corneal inflammation/ulcers or the organ failures shown in immune compromised individuals. These observations suggest that a persistent or non-lytic viral infection occurred in the experimental mice, which is typically observed in immune competent subjects with a CMV infection [1], [34]–[36]. To examine whether this was the case, we studied three kinds of blood vessel samples of all 48 mice by histological analysis. No lesion of lytic MCMV infection was found in blood vessel samples of viral infected mice, compared to their control counterparts. A representative result of histological examination in aorta section of mouse infected by MCMV vs. of mouse mock-infected has been shown in Fig. 2C and D.

**Figure 6:**
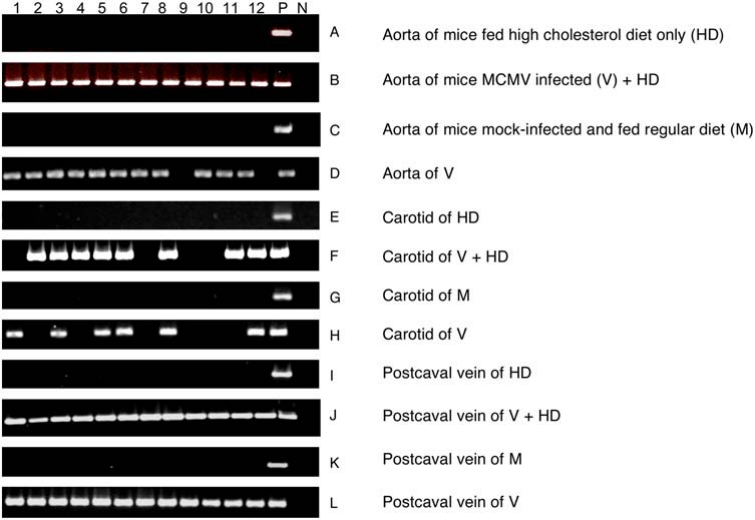
Detection of MCMV DNA in specimens of blood vessels. The viral immediate early gene-1 (IE-1) DNA was detected in blood vessel tissues of mice infected by MCMV. No viral IE-1 was detected in mice mock-infected and fed with either diet. The nested-PCR assay used two pairs of viral specific primers that amplify the MCMV IE-1 gene into a 310-bp DNA fragment. (A–L) Lanes 1–12, the blood vessel tissues of twelve mice from each experimental group. P, PCR positive control; N, PCR negative control. The positive rates of MCMV IE-1 in blood vessels of MCMV infected mice are shown in Table 2.

To confirm that CMV infection did occur in blood vessels of assay mice, we examined the expression of viral mRNA in mouse aorta in a separate experiment. At week 3 of infection, two mice in each of the four groups were examined for expression of MCMV IE1 mRNA in aortic tissues. The MCMV IE1 mRNA was detected only in viral infected mice but not in the control mice by RT-PCR assay (Fig. S2). We then examined expression of viral genomic DNA in blood vessels of experimental mice after 6 weeks of infection, as CMV is a DNA virus that replicates by amplifying its genomic DNA in the target cells even with a non-lytic infection. After examination of MCMV DNA in samples of aortic root, thoracic aorta, carotid, and post cava vein by PCR analysis, we found that the 23 out of 24 mice infected by MCMV had viral *ie1* DNA detected in the majority of aortas (96%) and in postcaval venous tissues (100%, Fig. 6, Table 2). No MCMV DNA was detected in mice that were mock-infected (Fig. 6, Table 2). These results support that CMV infection of blood vessel cells and expression of viral RNA and DNA played an important role in inducing the increase of blood pressure, potentially by altering expression of host cell genes that are involved in expression of pro-inflammatory cytokines, renin and Ang II, affecting vessel cell function and resulting in an increase of blood pressure.

**Table 2:** Detection of MCMV DNA in blood vessels.

	IE-1	Aorta		Carotid		Postcaval Vein	
		+	−	+	−	+	−
V+HD		12	0	8	4	12	0
HD		0	12	0	12	0	12
V		11	1	6	6	12	0
M		0	12	0	12	0	12

V+HD, mice infected by MCMV and fed with a high cholesterol diet. HD, mice fed with a high cholesterol diet. V, mice infected by MCMV and fed a regular diet. M, mice mock infected and fed a regular diet. Each group had 12 mice. The MCMV DNA IE-1 was examined in three types of vessel cells of all experimental mice. The numbers show the mice in each group positive and negative on the viral DNA.

### HCMV infection stimulates renin expression in vascular endothelial cells (EC)

CMV infection is highly cell and species specific, and HCMV infects only human cells but not mouse cells, and vice versa. To define whether HCMV infection is a risk factor inducing an increase of blood pressure in humans, we defined HCMV infection of both arterial and venous vascular endothelial cells, by an established *in vitro* culture system. In addition, expression of renin in EC is recognized as a key component of the local RAS [24]–[27].

**Figure 7:**
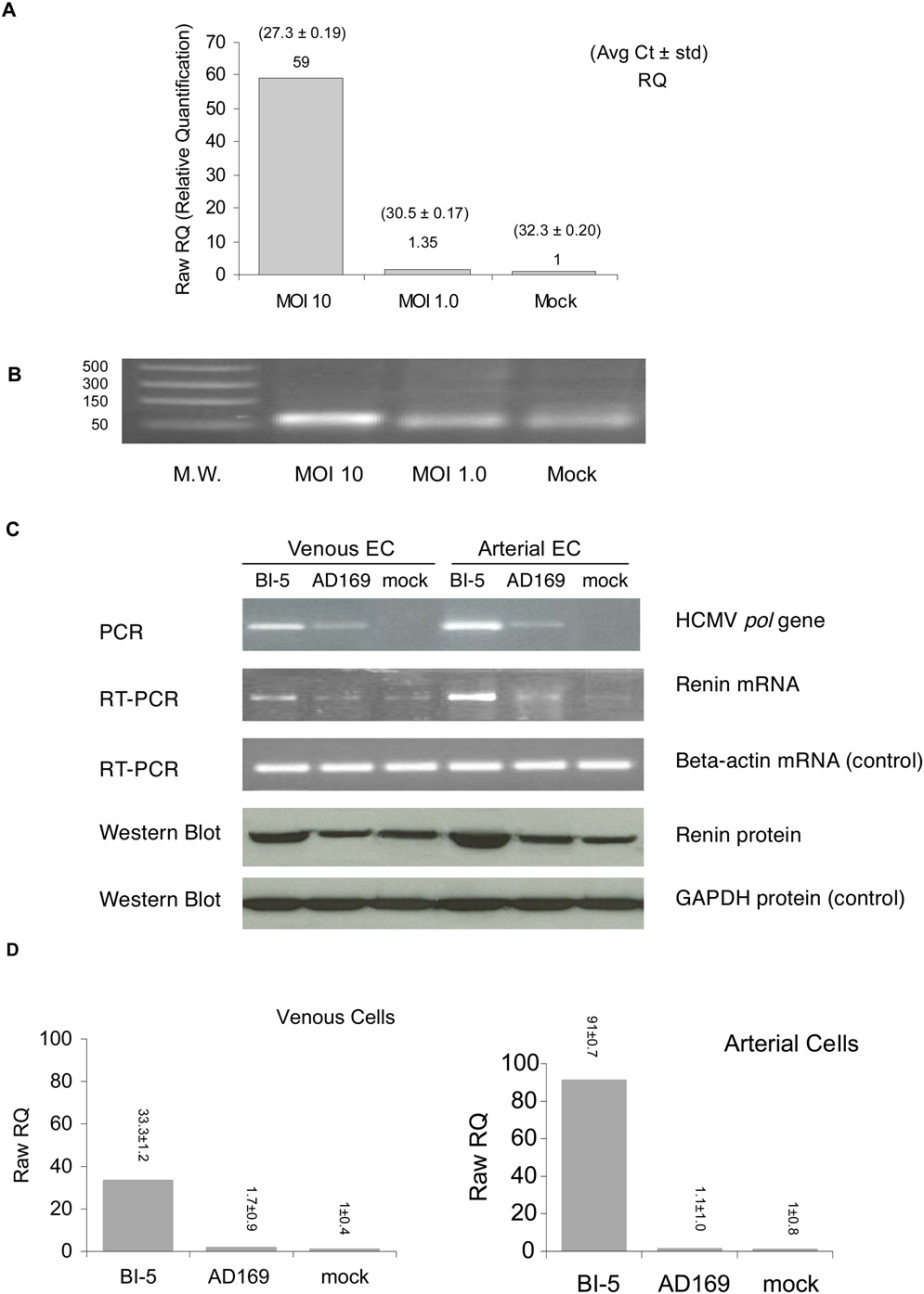
HCMV infection of human EC induced expression of renin. (A) HCMV infection induced expression of renin mRNA, determined by real time Q-RT-PCR assays. The expression of renin mRNA in cells showed correlation with an intensity of MOI 10>MOI 1, and cells with mock-infection (MOI 0) served as the base line (RQ = 1). (B) The products of Q-RT-PCR were confirmed by gel electrophoresis, showing that renin mRNA was highly expressed in cells infected with MOI 10, in contrast to the cells infected with MOI 1 and MOI 0 (mock infection). (C) HCMV infection induced renin expression in both venous (CRL-1730) and arterial (CRL-2472) EC, determined by PCR, RT-PCR and Western blot. To accurately measure the viral replication and renin expression, HCMV DNA polymerase (*pol*) gene and the renin mRNA levels in infected cells were determined by PCR and RT-PCR, respectively. Renin protein expressions were determined by Western blots in these cells. HCMV *pol* DNA was strongly detected in BI-5 (HCMV clinical isolate) infected venous and arterial cells. AD169, a lab strain known to be unable to grow in vascular endothelial cells, served as a viral infection control, which did not express viral *pol* gene nor induced renin expression. (D) HCMV BI-5 induced expression of renin mRNA in venous and arterial EC, determined by real time Q-RT-PCR assays.

Employing human umbilical vein (HUVEC, ATCC) and abdominal aorta endothelial cells (HAAE, ATCC), we found that HCMV infection stimulated expression of renin in EC in an infectious dose dependent manner, determined by expression of renin mRNA with Q-RT-PCR and expression of renin protein by Western blot (Fig. 7A–C). In contrast, the HCMV laboratory strain AD-169, which does not replicate in EC and expresses a minimal level of *pol*, did not induce renin expression (Fig. 7C and D). When EC were exposed to UV-inactivated HCMV, no increased expression of renin mRNA was detected (data not shown). Additionally, consistent with the findings of our mouse trial study, HCMV infection of both arterial and venous EC showed a non-lytic infection, determined by the absence of cell cytopathic effect (CPE) or viral plaque formation (Fig. S3, Fig. S4).

### HCMV persistently infects EC

**Figure 8:**
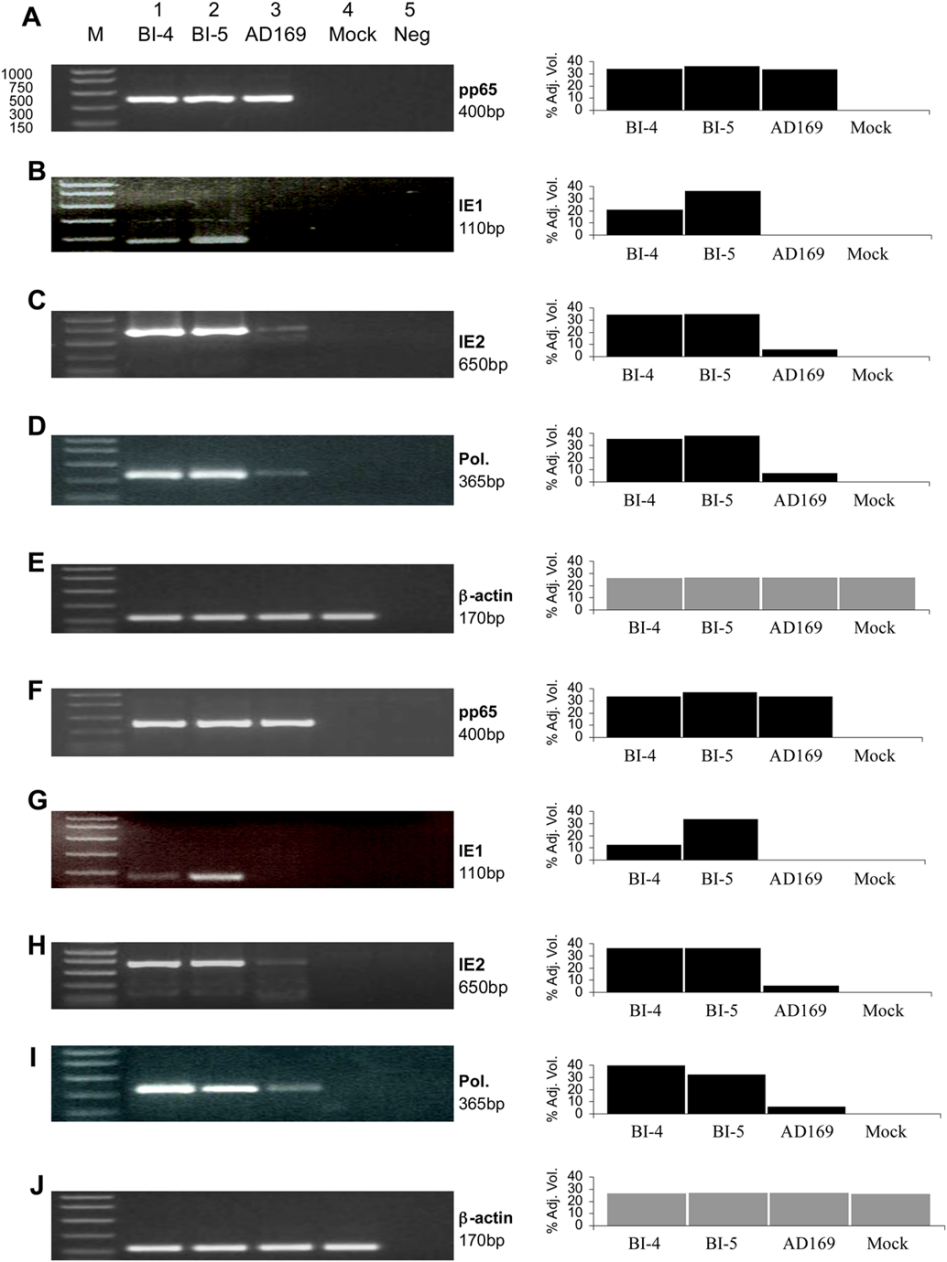
Detection of HCMV *ie1*, *ie2*, *pol*, and *pp65* mRNA in human EC. The viral gene expression was determined by RT-PCR assays at 14 days post infection. The pp65 gene expression served as the control. (A–E) HCMV RNA expression in infected umbilical vein cells (CRL-1730). (F–J) HCMV RNA expression in abdominal aorta cells (CRL-2472). Data show that HCMV clinical isolates, BI-4 and BI-5, expressed viral specific genes and persistently infected EC, whereas HCMV lab strain, AD-169, did not. The RT-PCR products examined by agarose gel are shown on the left panel, and by densitometer tracing are shown on the right panel.

To further clarify that HCMV infects EC in a persistent infection manner, we determined CMV early gene transcripts in EC by infecting cells with HCMV clinical isolates BI-4 and BI-5, in contrast to the lab strain AD169. Our data show that, compared to AD169, HCMV clinical isolates infected EC and expressed two key genes that were important for CMV replication, *ie2* and *pol*. The CMV mRNA of *ie2* and *pol* were detected in EC infected by HCMV clinical isolates (BI-4 and BI-5), but not by the lab strain (AD169) (Fig. 8). Furthermore, in a multi time-point CMV infection kinetic study, we defined that HCMV clinical isolates infected EC in a persistent infection manner, in which the copies of *pol* gene were quantitatively detected in cell culture supernatant, in contrast to the copy numbers of *pol* expression from AD169 known to be unable to replicate in EC (Fig. S5).

Taken together, our studies demonstrated that a persistent HCMV infection, not a lytic infection, plays a central role in vascular injury associated with HCMV. Specifically, a lytic infection is defined as evidence of viral replication that leads to a cytopathic effect, viral plague formation in tissue culture, and cell lysis with histologic evidence showing morphological cytopathic change in the mouse vessel walls. A persistent or non-lytic infection is defined as the evidence of viral gene expression, on DNA, RNA or protein levels, in the absence of viral cytopathic effect, cell lysis, or histological changes. We found that the results of cell culture assay are consistent with the observations of *in vivo* mouse model study, since no plaque formation was found in cell culture and no lytic lesions were observed in mouse aortas with microscopic analyses. Additionally, HCMV infection of EC further showed that CMV infection perturbed host cell gene expression, and the chronic inflammatory responses play a pivotal role in human cardiovascular diseases initiated by HCMV infection, specifically an increase of arterial blood pressure that was identified by our *in vivo* mouse model studies.

## Discussion

MCMV infection caused a significant increase of arterial blood pressure *in vivo*, independent of high cholesterol diet and atherosclerotic plaque formation in the mouse aorta (Fig. 1, Fig. 2, Tables 1 and 2). Additionally, we find that besides stimulating expression of inflammatory cytokines reported to increase blood pressure, CMV infection stimulated expression of renin, the first component of RAS, in both kidney cells and EC, in a dose-dependent manner (Fig. 4, Fig. 7). CMV infection also increased Ang II levels in mouse blood and artery tissues. Ang II has been known as the effector peptide of RAS that directly binds to blood vessels and induces vasoconstriction. Our experimental results show that CMV infection is a risk factor to cause cardiovascular diseases, specifically an increase of blood pressure or hypertension. A non-lytic CMV infection and the perturbed cellular gene expression, specifically the components of RAS, underlie a molecular mechanism by which CMV infection causes an increase of blood pressure.

In the *in vivo* mouse model study, we examined 48 mice of the same species with the same age, similar body weight, and evenly distributed male and female in 4 assay groups. We determined that infection by MCMV induced an increase of blood pressure, and compared this to the known risk factors of hypertension: high cholesterol diet and atherosclerosis. We employed a catheter method to measure the blood pressures of all assay mice, in the right carotid under anesthesia to obtain an accurate reading and reduce the pain of experimental animals. The mean values of blood pressure of the MCMV infected mice were compared to the control groups where the blood pressure was measured by the same technique and the same procedure. The experimental results show statistically significant differences between the MCMV infected groups and the control groups. Clearly, our *in vivo* mouse model study demonstrates that MCMV infection induced a significant increase of arterial blood pressure, independent of the other two known risk factors, high cholesterol diet and atherosclerosis, in mice that were immune competent and with normal lipid metabolic functions.

A high cholesterol diet induces an increase of blood pressure in association with weight gain [30]. MCMV infection did not contribute to a mouse weight gain (Fig. S1, *P*>0.05). The weights of two groups of C57BL/6J mice fed with a high cholesterol diet were greater than the two groups fed with a regular diet (*P*<0.05), and both high cholesterol diet groups, viral infected or not, had an increase of blood pressure (Fig. 1E). The high blood pressure in overweight mice suggests that obesity plays an important role in development of hypertension in mice fed with high cholesterol diet. Obesity causes hypercholesterolemia, which results in high blood viscosity, blood dynamic alterations, and vascular endothelial mechanistic injury. In our study, MCMV infected mice had significantly increased blood pressures compared to the mock-infected mice, in both diet groups (*P*<0.01 and *P*<0.05). The MCMV infected group did not weigh more than those in control groups fed with the same diet (Fig. S1, *P*>0.05). Thus, CMV infection did not contribute to the mouse weight gain, but caused an increase of blood pressure apparently via a change in expression of cellular genes, the over expression of inflammatory cytokines, renin and Ang II in the vascular system.

Mice were infected with MCMV via the intra-peritoneal injection. The viral IE-1 DNA was detected at week 6 post infection in 100% of aortas of infected mice fed high cholesterol diet, and in 92% of aortas of infected mice fed with a regular diet. We also examined MCMV DNA in other blood vessels. The majority of large vessels in the viral infected mice were positive for viral DNA, and MCMV has an affinity to infect vessel cells even though the port of viral infection is at the intra-peritoneum (Fig. 6, Table 2). Aorta and postcaval vein appear to be the main reservoirs of CMV. There was no significant difference in MCMV infection of vessel cells in mice fed with a high cholesterol diet compared to the mice fed with a regular diet (*P*>0.05). Nerheim et al., however, have reported that the active HCMV infection is enhanced in atherosclerotic blood vessels compared to atherosclerosis-free vascular equivalents, and this viral activity is restricted to the subpopulations of intimal and adventitial cells [37].

Renin is a step limiting protein of RAS. Increased RAS activity results in systemic hypertension and expression of ectopic anti-renin or anti-angiotensin molecules decreases hypertension [24]–[27]. We determined renin expression in both mouse and human cells after CMV infection, and find that CMV infection increased renin expression in cells in a MOI dependent manner (Fig. 4, Fig. 7).

Furthermore, in immune competent individuals, CMV causes persistent infections in certain types of cells, including EC. The course of infection is symptomless, and limited at the cell level. In our studies, all 4 groups of mice were followed for 10 weeks and no symptoms of CMV infection were observed. Examination of mouse specimens and infection of EC culture by CMV confirmed that a non-lytic infection occurred in blood vessel cells (Fig. 2, Fig. 8, Fig. S3, Fig. S4, Fig. S5).

In summary, CMV infection alone caused a significant increase of arterial blood pressure. Enhanced expression of pro-inflammatory cytokines, renin and Ang II underlies the pathogenic mechanism of an active CMV infection to increase blood pressure and aggravate atherosclerosis. Thus, control of CMV infection to restrict development of hypertension and atherosclerosis may provide a new strategy to prevent cardiovascular diseases associated with HCMV infection.

## Materials and Methods

### Viruses

Mouse cytomegalovirus (MCMV, strain smith MSGV), was purchased from American Type Culture Collection (ATTC, VR-1399). MCMV stock was prepared by infection of mouse embryo fibroblasts (ATCC CRL-1404) and collection of the culture supernatant after cytopathic effect (CPE) was appeared on the cell monolayer. The supernatants were centrifuged at 1,400 rpm for 15 min to get rid of the cellular debris, and supernatants were stored at −80°C in aliquots. The titer of the viral stock was determined by the standard plaque assay [38]. The clinical isolates of human cytomegalovirus (HCMV), BI-4 and BI-5, were obtained from patient samples as previously described and generously provided by Dr. Lurain (Rush University Medical Center, Chicago, IL) [39]. The viral pool of laboratory strain AD169_ATCC_ was prepared from human fetal lung fibroblast (MRC-5, ATCC) cell culture. Viruses were titrated on human foreskin cells (HF, ATCC) following a consensus HCMV titration protocol [40].

### Study subjects

Two-week-old C57BL/6J mice (n = 48) were obtained from the Jackson Laboratory (Bar Harbor, Maine). Experiments employed 2-week-old mice, housed in our animal facility with 3 mice (the same gender) in one cage. Mice had free access to tap water, and fed with regular or atherogenic commercial diet (1.25% cholesterol, 0.5% cholic acid and 15% fat), purchased from the Jackson laboratory. All animal experiments were performed in accordance with National Institutes of Health guidelines. Protocols were approved by the Animal Care and Use Committees of Beth Israel Deaconess Medical Center and Harvard Medical School.

### Experimental design of the mouse trial study

As is shown in Table 3.

**Table 3:** Experimental design of the mouse trial study.

Group	Quantity	Week 0	Week 4	*Week 10
MCMV+Regular diet (V)	12 (F6+M6)	Measure weight and begin diet	MCMV infection, 300,000 pfu/1ml/mouse, Intraperitoneal (IP)	Measure weight, blood pressure, sacrifice mice and collect samples and tissues
Mock infection+regular diet (M)	12 (F6+M6)	Same as above	Mock infection, 1ml PBS/mouse (IP)	Same as above
MCMV+High cholesterol diet (V+HD)	12 (F6+M6)	Same as above	MCMV infection, 300,000 pfu/1ml/mouse, Intraperitoneal (IP)	Same as above
High cholesterol diet (HD)	12 (F6+M6)	Same as above	Mock infection, 1ml PBS/mouse (IP)	Same as above

*: Week 6 post-infection. F: female. M: male. MCMV was diluted to above concentration with phosphate buffered saline (PBS).

### Measurement of mouse blood pressure and collection of specimen

Six weeks after MCMV infection (at week 10 of the experiment), mice were anesthetized with pentobarbital sodium (40 mg/kg, IP). The carotid artery was isolated and cannulated with a 1.4-Fr (SPR-671) high-fidelity microtip transducer catheter connected to a data acquisition system (PowerLab ML820, ADInstrument, Colorado Springs) through a pressure interface unit (Millar Instrument, Transducer Balance, TCB 600). The microtip catheter was advanced into the carotid artery and carotid blood pressure was recorded. The blood pressure data were collected and analyzed using a Chart v4.1.2 software of AD-instrument [28],[29]. Then the end of carotid artery toward the heart was ligated and the arteries were cut between two ligations (one end toward heart and the other end toward brain). The fragment of carotid artery was washed in PBS one time, and protein in O.C.T medium then stored in liquid nitrogen. Following that, 200∼400 µl of blood was collected from the jugular vein and spun at 7,000 rpm for 10 minutes to remove cells. The plasma was stored at −80°C. Aorta and postcaval vein were also collected, washed with PBS to remove blood in vessels, and kept in liquid nitrogen. The whole aorta was divided into three parts, of which the root was used to prepare frozen sections and H&E stain for pathology; the upper and lower chest fragment (about 0.5 to 1 cm) were used for RNA extraction/gene analysis and DNA extraction/PCR assay, respectively.

### Statistical analysis

Comparisons between 2 experimental groups were processed by Student's *t* test (2-tailed) and Chisquare (*χ* ^2^) Test. *P* value≤0.05 was considered statistically significant for a difference between groups.

### Immunofluorescent staining expression of renin and MCMV antigen

The mouse kidney intraparenchymal cell line CRL-2193 (As4.1) was purchased from ATCC. Cells have a basal level of intracellularly secreted prorenin. To examine the effect of MCMV infection on renin expression, the cells were infected with MCMV and then allowed to grow in chambered slides for 5 days. The culture slices were rinsed twice with PBS, and cells were fixed by 10% formaldehyde and permeabilized in 0.2% triton-X-100 in PBS. Cells were incubated with 5% BSA (blocking reagent), and then incubated further in PBS/5% BSA with 1∶200 diluted primary antibodies mouse anti-renin (Serotec), and mouse anti-murine CMV (Express Biotech). After washing, the cells were incubated with 1∶300 diluted FITC-coupled anti-mouse IgG (Delta Biolabs) or TRITC-coupled anti-mouse IgG (Invitrogen). The cells were observed under a fluorescence microscope (Olympus, DP70) after added mounting medium containing DAPI.

### Angiotensin II ELISA

Angiotensin II ELISA kit was purchased from Bachem (Torrance, CA) and sample testing followed manufacturer's instructions. The mouse serum was prepared as described in the sample collection section. For detection of Ang II in vessel samples, 0.2 inch of aorta fragment of 3 randomly selected mice from each group were diced and put in a 2-ml tube with 470 µl of lysis buffer, respectively (20 mM HEPES, pH 7.5, 150 mM NaCl, 1% Triton X-100, 0.1% SDS, 1 mM EDTA, 1 mM DTT, 2 tablets of Roche protease inhibitor/50 ml). The tissues were then manually disrupted in lysis buffer with a small pestle, respectively. The solution was then incubated in 55°C overnight. After centrifugation, the supernatant of cell lysate was collected from each tube and then used for detection of Ang II by ELISA.

### Nested-PCR test of MCMV DNA in blood vessel cells

The vessel tissues were disrupted with a pestle in the tissue digestive buffer (50 mM Tris-HCl, 100 mM NaCl, 5 mM EDTA, 1% SDS, 10 mg/ml Protease K) and digested at 50°C overnight. After phenol and chloroform extraction, the DNA pellet was washed with 70% alcohol, dissolved in 100 µl ddH_2_O, and kept in −80°C till to PCR. The Nested PCR primers that amplify the MCMV immediate early gene-1 (IE-1) are as previously described [41]–[43].

### Real-time quantitative RT-PCR

The Real-Time RT-PCR was performed with Applied Biosystems 7300 Fast Real-Time PCR system using TaqMan probe and primer based techniques. The probe and primers are specific to cDNA of mouse and human renin, respectively, and were designed and purchased from Applied Biosystems with the assay ID numbers 433734 (mouse) and 418327 (human). The Relative Quantification (RQ) experiments were performed following the RQ protocol, and results of RQ experiments are reported as the normalized reporter dye fluorescence (Rn) as a function of cycle number for each sample, compared to the internal control.

### Cells and HCMV infection

Umbilical vein endothelial cell line (CRL-1730) and abdominal aorta endothelial cell line (CRL-2472) were purchased from ATCC. The log growth cells were split into 6-well plates two days before CMV infection. After forming the monolayer, cells were infected at multiplicities of infection (MOI) of 10 from each virus, and the unadsorbed virus was washed off with PBS after incubation. The supernatant of 0.5 ml from each culture was collected at day 0, 3, 7, 10, and 14 post infection and replaced back with 0.5 ml of fresh medium. Virus-induced cytolytic effect was examined at each time point with the phase contrast microscope. The COBAS amplicor CMV monitor test was adapted to measure the HCMV *pol* DNA copy numbers in culture supernatants, using the COBAS AMPLICOR Analyzer (Roche Molecular Systems). This quantitative DNA PCR assay specifically detects a 365-base fragment in HCMV pol gene that is not homologous to other herpes viruses. The amplification kit was purchased from Roche Diagnostic Systems, Inc. (Branchburg, NJ), and specimen preparation, PCR amplification and HCMV DNA quantification followed the manufacturer's instructions.

### RT-PCR examining expression of pp65, IE1, IE2, and pol mRNA in infected cells

The RT-PCR primers were designed based on HCMV *pp65*, *ie1* and *ie2* (IE 55/86) exon sequences, respectively [42],[43]. HCMV *pp65* forward primer: 5′-CACCTGTCACCGCTGCTATATT TGC-3′; reverse primer: 5′-CACCACGCAGCGGCCCTTGATGTTT-3′. The *ie1* forward primers: 5′-CTTAATACAAGCCATCCACA-3′; reverse primer: 5′-TAGATAAGGTTCATGAGCCT-3′. The *ie2* forward primer: 5′-GCACACCCAACGTGCAGACTCGGC-3; reverse primer: 5′-TGGCTGCCTCGA TGGCCAGGCTC-3′. The primer set for amplification of HCMV *pol* is the same as that used in COBAS assay except the reverse transcription process, and amplifies a 365-base fragment in HCMV *pol* gene that is not homologous to other herpes viruses. RT-PCR was performed using OneStep RT-PCR kit (Qiagen, Valencia, CA) according to manufacturer's instructions. The reaction mixtures were incubated in a thermocycler 9600 under the following conditions: 50°C for 30 minutes, 95°C for 15 minutes, and then 94°C for 30 second, 55°C for 30 second, and 72°C for 1 minute, total 35 cycles, finally 72°C for 10 minutes.

### Extraction of total RNA from infected cells

Cells from each two wells (duplicate) were scraped and RNA extraction and purification were followed the protocol of purification of total RNA from animal tissues (RNeasy Mini Kit, Qiagen Company). The On-Column DNase digestion was applied to remove remaining DNA from the RNA samples (RNase-Free DNase Set, Qiagen Company). The RNA concentration was measured at the absorbance of O.D. 260, and the purity was further examined by electrophoresis.

## Supporting Information

Figure S1 Mouse weight change before and after experiment. Weight changes of C57BL/6J mice before and after experiment. HD, mean value of weights in the group of mice mock-infected and fed a high cholesterol diet only. V+HD, MCMV-infected and fed a high cholesterol diet. M, mock-infected and fed a regular diet only. V, MCMV-infected and fed a regular diet.
(0.32 MB TIF)

Figure S2 Detection of MCMV IE1 mRNA in mouse aorta. MCMV IE1 mRNA in murine aortas from 4 groups of mice tested by RT-PCR. M, Molecular weight marker. Lanes 1–2: No MCMV IE1 was detected in RNA samples of 2 mice in regular diet group. Lanes 3–4: MCMV IE1 mRNA was detected in RNA samples of 2 mice in MCMV infection plus regular diet group. Lanes 5–6: No MCMV IE1 was detected in RNA samples of 2 mice in high cholesterol diet group. Lanes 7–8: MCMV IE1 was detected in RNA samples of 2 mice in MCMV infection plus high cholesterol diet group. P, RT-PCR positive control. N, RT-PCR negative control. Extraction of total RNA was the same as described previously. RT-PCR primers, Forward: 5′ CCTCGAGTCTGGAACCGAAA 3′; reverse: 5′ TACAGGACAAC AGAACGCTC 3′. The total cellular RNA was reverse-transcribed and cDNA product was amplified by PCR at 50°C 30 minutes, 95°C 15 minutes, and then 94°C 30 seconds, 55°C 30 seconds, and 72°C 1 minute for 35 cycles, with a final extension at 72°C 10 minutes.
(0.41 MB TIF)

Figure S3 Non-lytic infection of venous EC by HCMV BI-4, BI-5 and AD169. The morphology of CRL-1730 cells after HCMV infection. (A) Day 1 post mock-infection. (B) Day 1 post BI-4 infection. (C) Day 1 post BI-5 infection. (D) Day 1 post AD169 infection. (E) Day 14 post mock-infection. (F) Day 14 post BI-4 infection. (G) Day 14 post BI-5 infection. (H) Day 14 post AD169 infection.
(1.61 MB TIF)

Figure S4 Non-lytic infection of arterial EC by HCMV BI-4, BI-5, and AD169. The morphology of CRL-2472 cells after HCMV infection. (A) Day 1 post mock-infection. (B) Day 1 post BI-4 infection. (C) Day 1 post BI-5 infection. (D) Day 1 post AD169 infection. (E) Day 14 post mock-infection. (F) Day 14 post BI-4 infection. (G) Day 14 post BI-5 infection. (H) Day 14 post AD169 infection.
(2.2 MB TIF)

Figure S5 HCMV persistently infects EC. Kinetic detection of HCMV infection of EC by expression of *pol* gene. (A) Detection of *pol* gene expression in venous EC (CRL-1730) infected by BI-4, BI-5 and AD169. (B) Detection of *pol* gene expression in arterial EC (CRL-2472) infected with BI-4, BI-5 and AD169. The EC were infected by clinical isolates BI-4, BI-5 and lab strain AD-169 at an MOI of 10, respectively. The supernatants of viral infected cultures were harvested at each time point, and HCMV *pol* DNA copy numbers were determined by the quantitative HCMV DNA-PCR assays (COBAS). Results shown were from a representative experiment of three performed, and data show that HCMV clinical isolates persistently infected EC, in contrast to the lab strain AD169.
(0.54 MB TIF)

Table S1 Blood pressures of C57BL/6J mice at week 4 and 10 of experiment (*base line). *The base line of blood pressure was measured in the right carotid of each mouse at week 4 before the MCMV infection, and mice were randomly selected from each group. These mice were treated the same as the other mice in the rest of experiment. At week 10 of the experiment, the blood pressures of these mice were measured again at the left carotids. The ABp values of these mice at week 10 were not included in the mean value of ABp measurement from each of the four experimental groups that consisted of 12 mice in each group.
(0.03 MB DOC)
