# Supplementary figures and images for: Cytomegalovirus Infection Causes an Increase of Arterial Blood Pressure
Source: PLoS Pathog. 2009 May 15;5(5):e1000427. doi: 10.1371/journal.ppat.1000427 (PMC2673691; doi:10.1371/journal.ppat.1000427)

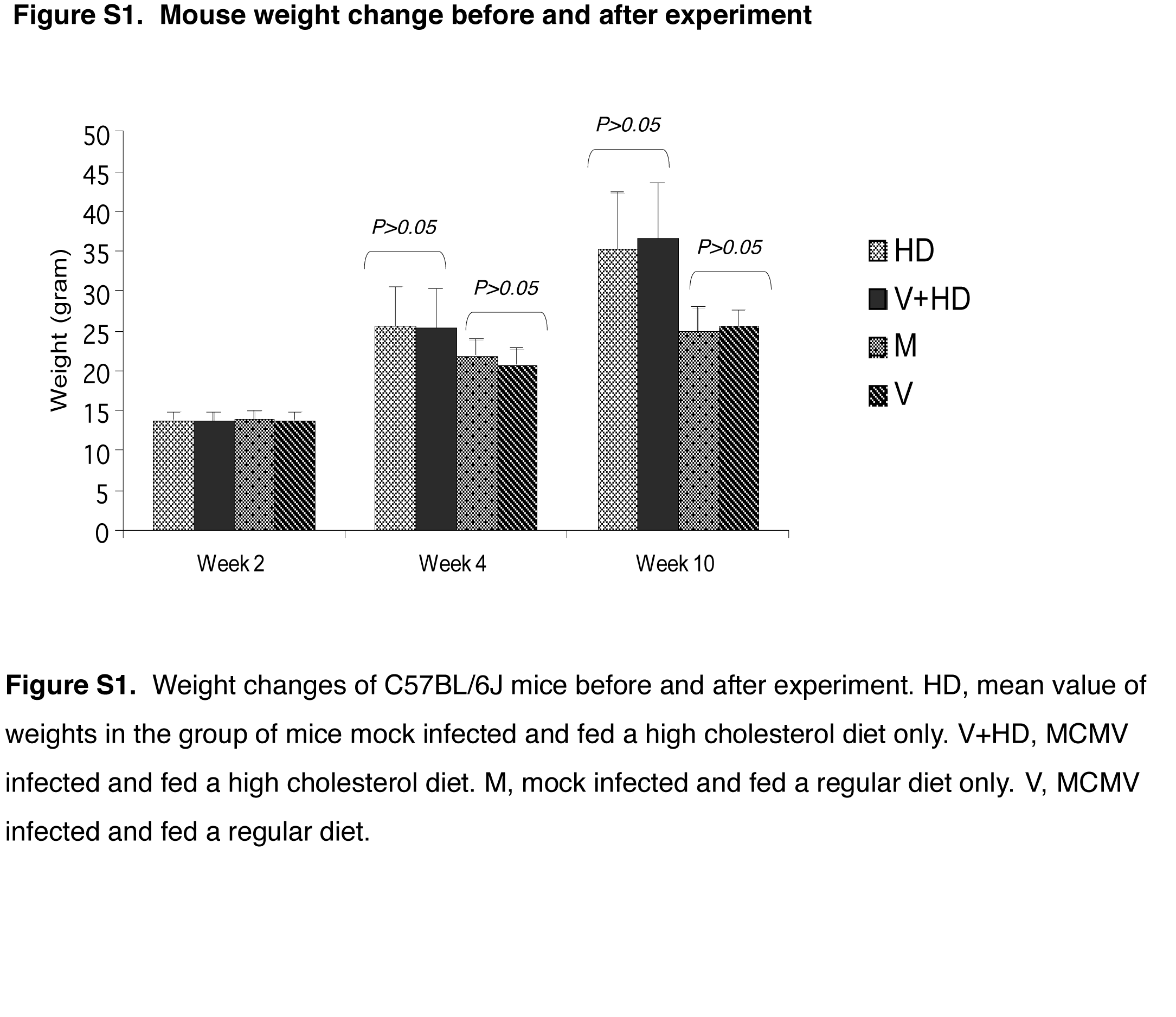

Supplement: Figure S1 — Mouse weight change before and after experiment. Weight changes of C57BL/6J mice before and after experiment. HD, mean value of weights in the group of mice mock-infected and fed a high cholesterol diet only. V+HD, MCMV-infected and fed a high cholesterol diet. M, mock-infected and fed a regular diet only. V, MCMV-infected and fed a regular diet. (0.32 MB TIF) [file ppat.1000427.s001.tif]

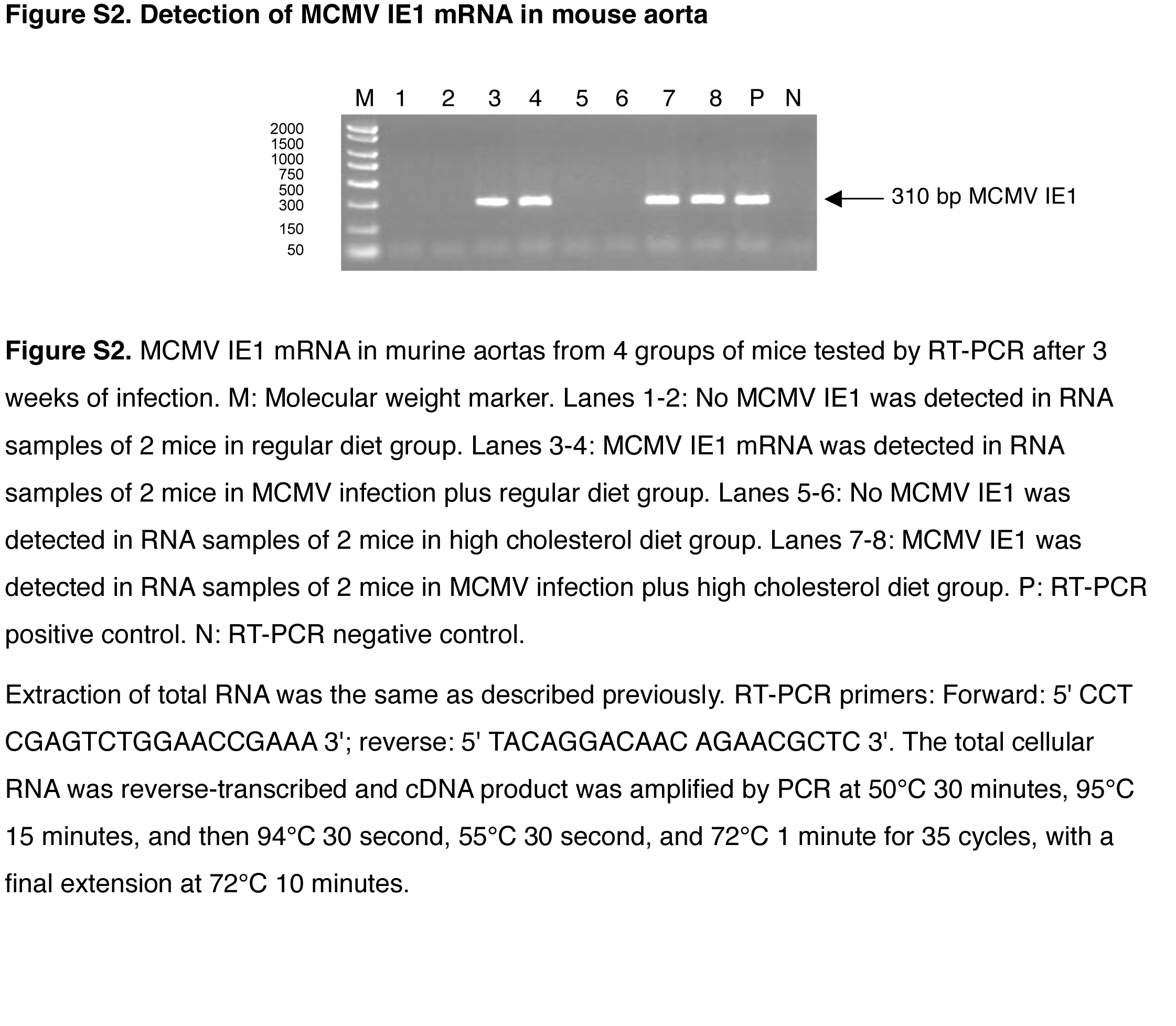

Supplement: Figure S2 — Detection of MCMV IE1 mRNA in mouse aorta. MCMV IE1 mRNA in murine aortas from 4 groups of mice tested by RT-PCR. M, Molecular weight marker. Lanes 1–2: No MCMV IE1 was detected in RNA samples of 2 mice in regular diet group. Lanes 3–4: MCMV IE1 mRNA was detected in RNA samples of 2 mice in MCMV infection plus regular diet group. Lanes 5–6: No MCMV IE1 was detected in RNA samples of 2 mice in high cholesterol diet group. Lanes 7–8: MCMV IE1 was detected in RNA samples of 2 mice in MCMV infection plus high cholesterol diet group. P, RT-PCR positive control. N, RT-PCR negative control. Extraction of total RNA was the same as described previously. RT-PCR primers, Forward: 5′ CCTCGAGTCTGGAACCGAAA 3′; reverse: 5′ TACAGGACAAC AGAACGCTC 3′. The total cellular RNA was reverse-transcribed and cDNA product was amplified by PCR at 50°C 30 minutes, 95°C 15 minutes, and then 94°C 30 seconds, 55°C 30 seconds, and 72°C 1 minute for 35 cycles, with a final extension at 72°C 10 minutes. (0.41 MB TIF) [file ppat.1000427.s002.tif]

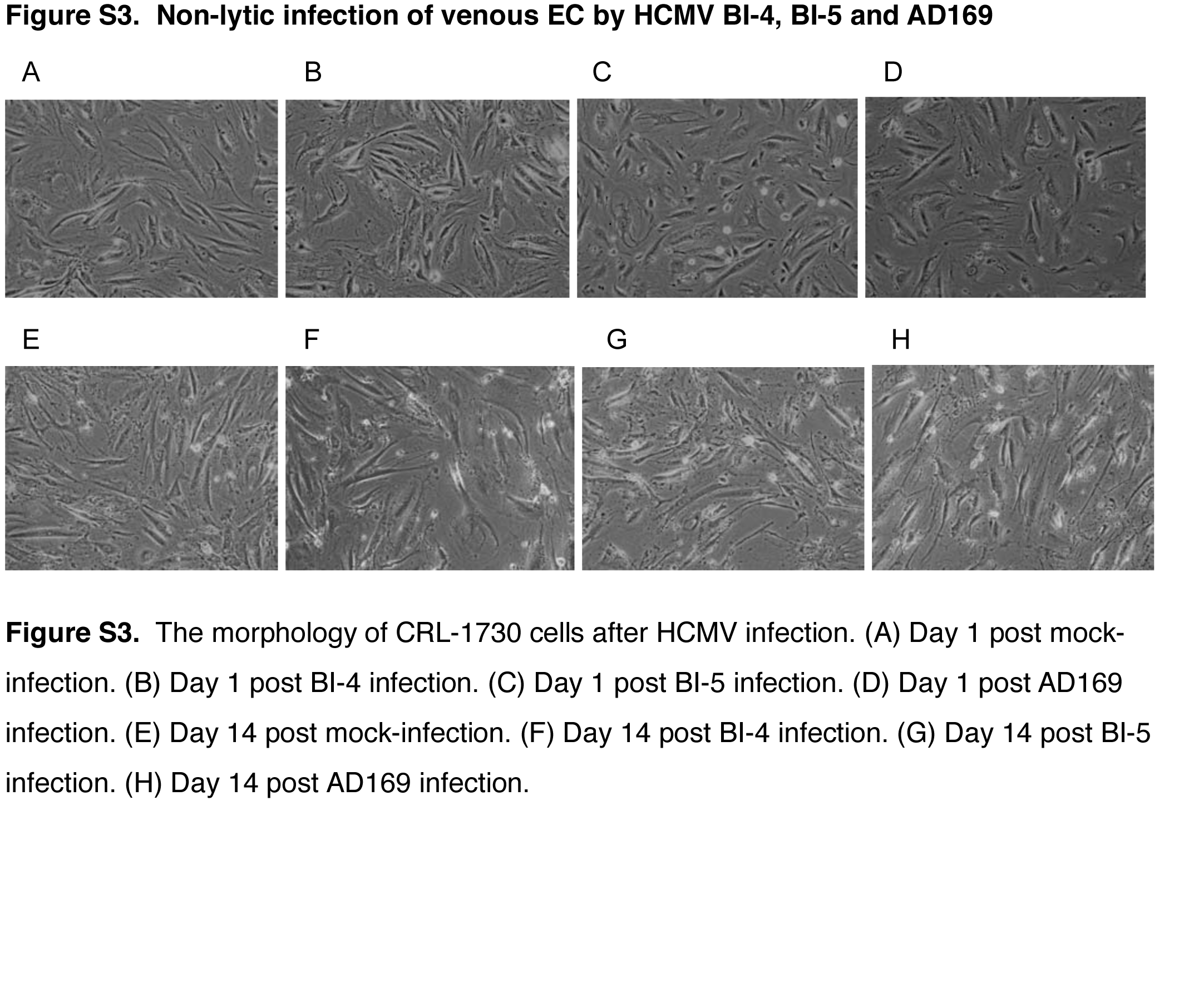

Supplement: Figure S3 — Non-lytic infection of venous EC by HCMV BI-4, BI-5 and AD169. The morphology of CRL-1730 cells after HCMV infection. (A) Day 1 post mock-infection. (B) Day 1 post BI-4 infection. (C) Day 1 post BI-5 infection. (D) Day 1 post AD169 infection. (E) Day 14 post mock-infection. (F) Day 14 post BI-4 infection. (G) Day 14 post BI-5 infection. (H) Day 14 post AD169 infection. (1.61 MB TIF) [file ppat.1000427.s003.tif]

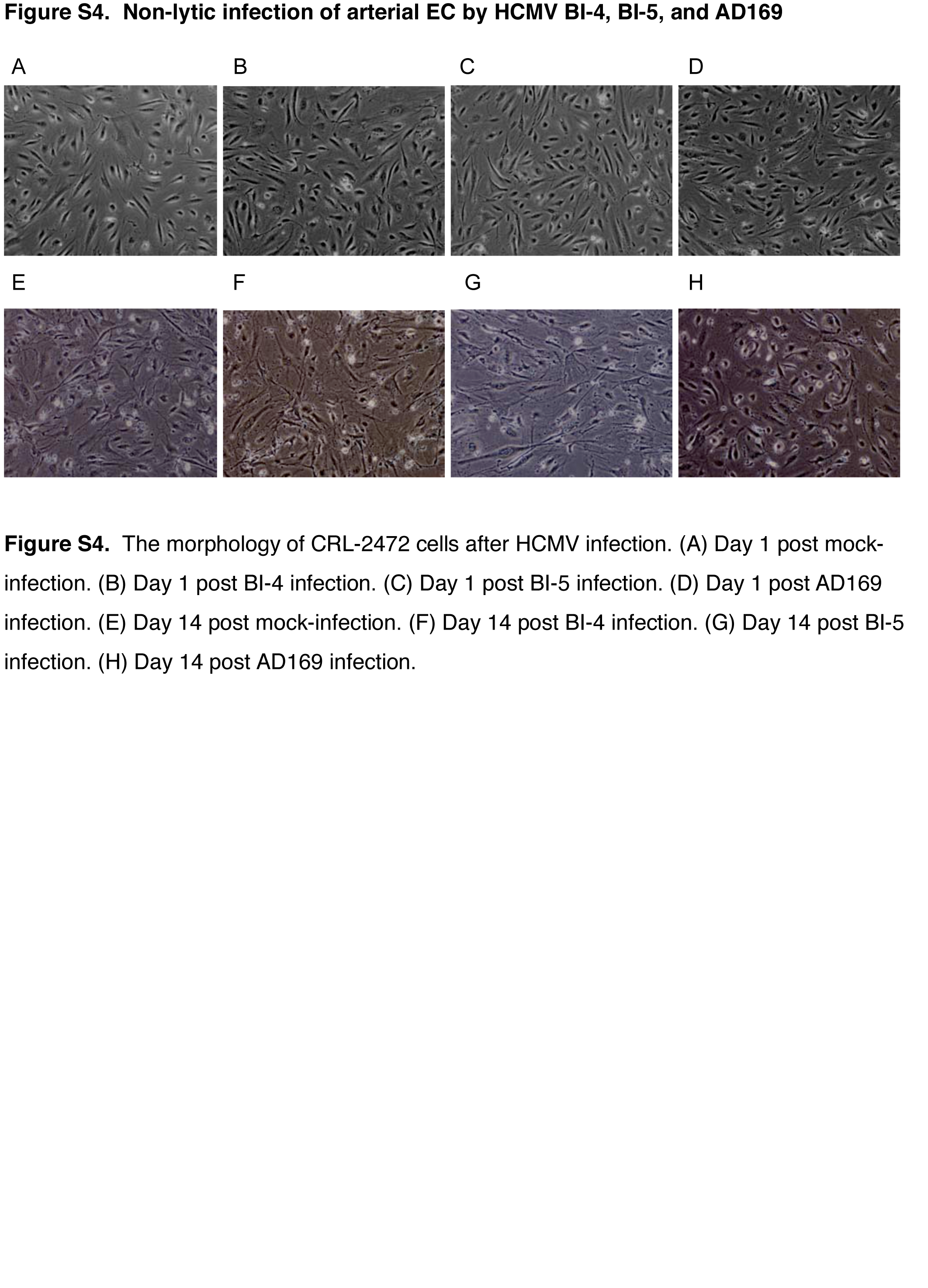

Supplement: Figure S4 — Non-lytic infection of arterial EC by HCMV BI-4, BI-5, and AD169. The morphology of CRL-2472 cells after HCMV infection. (A) Day 1 post mock-infection. (B) Day 1 post BI-4 infection. (C) Day 1 post BI-5 infection. (D) Day 1 post AD169 infection. (E) Day 14 post mock-infection. (F) Day 14 post BI-4 infection. (G) Day 14 post BI-5 infection. (H) Day 14 post AD169 infection. (2.2 MB TIF) [file ppat.1000427.s004.tif]

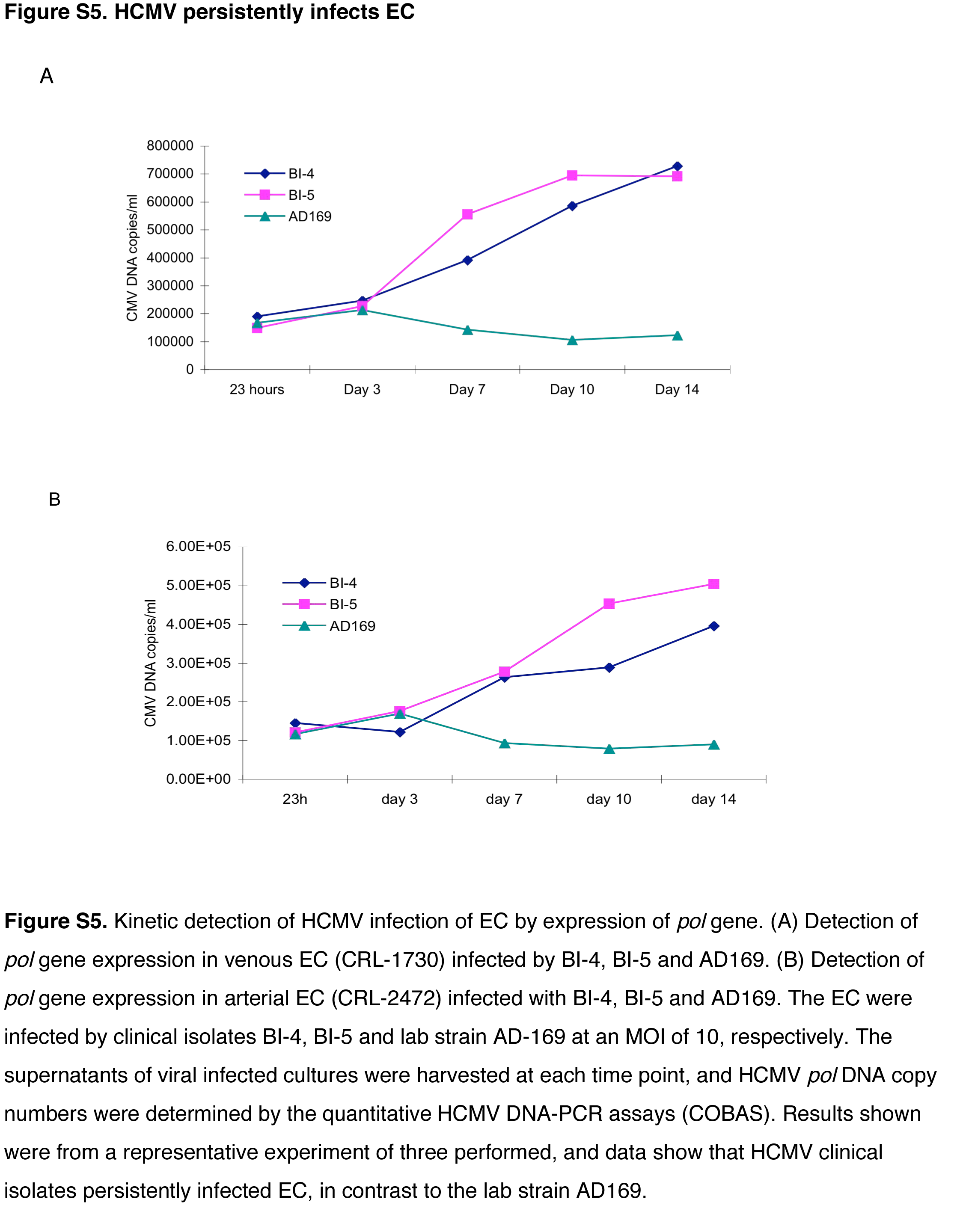

Supplement: Figure S5 — HCMV persistently infects EC. Kinetic detection of HCMV infection of EC by expression of pol gene. (A) Detection of pol gene expression in venous EC (CRL-1730) infected by BI-4, BI-5 and AD169. (B) Detection of pol gene expression in arterial EC (CRL-2472) infected with BI-4, BI-5 and AD169. The EC were infected by clinical isolates BI-4, BI-5 and lab strain AD-169 at an MOI of 10, respectively. The supernatants of viral infected cultures were harvested at each time point, and HCMV pol DNA copy numbers were determined by the quantitative HCMV DNA-PCR assays (COBAS). Results shown were from a representative experiment of three performed, and data show that HCMV clinical isolates persistently infected EC, in contrast to the lab strain AD169. (0.54 MB TIF) [file ppat.1000427.s005.tif]
