# Supplementary material for: Cytomegalovirus Infection Causes an Increase of Arterial Blood Pressure
Source: PLoS Pathog. 2009 May 15;5(5):e1000427. doi: 10.1371/journal.ppat.1000427 (PMC2673691; doi:10.1371/journal.ppat.1000427)
Supplement: Table S1 — Blood pressures of C57BL/6J mice at week 4 and 10 of experiment (*base line). *The base line of blood pressure was measured in the right carotid of each mouse at week 4 before the MCMV infection, and mice were randomly selected from each group. These mice were treated the same as the other mice in the rest of experiment. At week 10 of the experiment, the blood pressures of these mice were measured again at the left carotids. The ABp values of these mice at week 10 were not included in the mean value of ABp measurement from each of the four experimental groups that consisted of 12 mice in each group. (0.03 MB DOC) [file ppat.1000427.s006.doc]

**Table S1.** Blood pressures of C57BL/6J mice at week 4 and 10 of experiment (*base lne)

| **Mouse** | **Treatment** | **ABp (mmHg)** | | | |
| --- | --- | --- | --- | --- | --- |
|  |  | **Systolic** | | **Diastolic** | |
|  |  | **Week 4** | **Week 10** | **Week 4** | **Week10** |
| 1 | Mock | 80.42 | 83.48 | 50.07 | 49.90 |
| 2 | MCMV | 80.16 | 89.73 | 49.41 | 56.10 |
| 3 | HD | 85.70 | 101.41 | 54.23 | 70.60 |
| 4 | HD-MCMV | 84.19 | 140.77 | 56.84 | 96.94 |

*****The base line of blood pressure was measured in the right carotid of each mouse at week 4 before the MCMV infection, and mice were randomly selected from each group. These mice were treated the same as the other mice in the rest of experiment. At week 10 of the experiment, the blood pressures of these mice were measured again at the left carotids. The ABp values of these mice at week 10 were not included in the mean value of ABp measurement from each of the four experimental groups that consisted of 12 mice in each group.
